# The CUIDA Chagas Project: towards the elimination of congenital transmission of Chagas disease in Bolivia, Brazil, Colombia, and Paraguay

**DOI:** 10.1590/0037-8682-0171-2021

**Published:** 2022-04-29

**Authors:** Andréa Silvestre de Sousa, Debbie Vermeij, Gabriel Parra-Henao, Vidalia Lesmo, Evelin Fortún Fernández, José Jorge Chura Aruni, Fernanda de Souza Nogueira Sardinha Mendes, Laura C. Bohorquez, Alejandro O. Luquetti

**Affiliations:** 1 Fundação Oswaldo Cruz, Instituto Nacional de Infectologia Evandro Chagas, Rio de Janeiro, RJ, Brasil.; 2 Universidade Federal do Rio de Janeiro, Faculdade de Medicina, Rio de Janeiro, RJ, Brasil.; 3 Instituto Nacional da Saúde (INS), Bogotá, Colombia.; 4 Servicio Nacional de Erradicación del Paludismo (SENEPA), Assunción, Paraguay.; 5 Instituto Nacional de Laboratórios de Saúde Dr. Néstor Morales Villazón (INLASA), La Paz, do Estado Plurinacional de Bolívia.; 6 FIND, Bogota, Colombia.; 7 Universidade Federal de Goiás, Hospital das Clínicas, Núcleo de Estudos da Doença de Chagas, Goiânia, GO, Brasil.

**Keywords:** Trypanosoma cruzi, Chagas disease, Congenital infection, Mother to child transmission, Primary health care, Vulnerable populations

## Abstract

**Background:**

Mother-to-child transmission of Chagas disease (CD) has become a relevant problem in both endemic and non-endemic areas.

**Methods:**

Description of the CUIDA Chagas Project - Communities United for Innovation, Development and Attention for Chagas disease’.

**Results:**

Through innovative and strategic research, this project will provide improved diagnostic and treatment options as well as replicable implementation models that are adaptable to different contexts.

**Conclusions:**

By integrating test, treat and care actions for CD into primary health care practices, the burden of CD on people and health systems may be significantly reduced.

Chagas disease (CD), also known as American trypanosomiasis, is a neglected tropical disease (NTD) caused by the protozoan parasite *Trypanosoma cruzi* (*T. cruzi*) with a high morbimortality burden^1^. As a potentially life-threatening illness, an estimated 30-40% of untreated infected individuals will develop severe medical problems over the course of their lives, including cardiac alterations, digestive manifestations, and neurological or associated alterations^2^. 

Despite the high morbidity and mortality and significant associated economic burden, only about 7% of people with CD have been diagnosed, and approximately 1% receive etiological treatment^3^. With the relative success of vector and transfusion transmission control measures, mother-to-child transmission (MCT) has become proportionally more relevant, in addition to being the main source of new cases in non-endemic countries^1^ ^,^ ^2^.

An estimated 6 to 7 million people worldwide are infected with *T. cruzi* parasite, of which the large majority resides in Latin America (LA), and every year over 10,000 CD-related deaths are reported^1^. Of the aforementioned number of infected people, approximately 1.12 million are women of childbearing age (WCBA)^4^, and the congenital transmission rate approaches 5%, with higher rates in high-risk endemic areas^5^. Poor and vulnerable populations in endemic countries are especially affected, due to a multitude of barriers that prevent them from obtaining the assistance they need^2^ ^,^ ^6^. 

The incidence of congenital *T. cruzi* infection is estimated to be 8,000-15,000 cases per year in LA^7^; however, as maternal and child health (MCH) services do not routinely screen mothers or newborns for CD in most endemic areas, prevalence in pregnant women and newborns may be underestimated. Congenital infections could perpetuate CD indefinitely, even in non-endemic countries^8^.

Although access to prenatal care and childbirth for pregnant women is high in the Americas, problems persist in relation to screening for congenital diseases, which requires the integration of surveillance actions with those of health care^4^. Recommended interventions to control congenital CD are available in all endemic countries; however, data on health service coverage are limited. Based on the limited information provided by countries to the Pan American Health Organization (PAHO), screening for CD in pregnant women varies widely from just over 5% to almost 60% among the few countries that have reported it^4^ ^,^ ^9^. 

Some international strategies and action plans, such as the elimination of mother-to-child transmission (EMTCT) initiative from PAHO and the roadmap for NTDs from the World Health Organization (WHO), have been launched to achieve the elimination of congenital transmission of CD, but many countries do not yet have proper programs in place^4^. Scarcity of diagnostic tools and treatment options, poor treatment adherence, a lack of knowledge and understanding among health providers and people at risk, socio-economic vulnerabilities of endemic areas, and low social mobilization only aggravate the problem^6^, as does the COVID-19 pandemic^10^.

The CUIDA Chagas project, launched on April 14, 2021, the World Chagas Day, aims to contribute to the elimination of congenital transmission of CD in Latin America. This initiative is funded by Unitaid and the Brazilian Ministry of Health (MoH) and will be implemented in four endemic countries. 

The CUIDA Chagas consortium consists of key players in the public health landscape from Bolivia, Brazil, Colombia and Paraguay, and is endorsed by the MoH of each country. Countries were selected based on extensive consultations with key stakeholders and factors beyond disease burden, such as replicability, potential impact on regional sustainable change, and political commitment. Led by Brazil’s Fiotec/Fiocruz, the consortium includes organizations, such as Instituto Nacional de Laboratorios de Salud “Néstor Morales Villazón” (INLASA) from Bolivia, Instituto Nacional de Salud (INS) from Colombia, Servicio Nacional de Erradicación del Paludismo (SENEPA) from Paraguay, and the international non-governmental organization FIND, the global alliance for diagnostics 

Through a combination of implementation and innovation research, this project aims to present a comprehensive and integrated approach to address its goal of contributing to the elimination of congenital transmission of CD. This goal will be achieved through two outcomes: (1) increased access to and demand for effective diagnostics, treatment, and care for CD, and (2) improved diagnostic algorithms and treatment options validated, and access conditions ensured. These outcomes will be achieved through specifically designed activities under five outputs: (1) evidence generated on effective test, treat, and care approaches through implementation research; (2) community and civil society engaged at local, national and regional levels to increase demand for services and advocate for integration of recommended approaches for CD in policies, strategies and plans; (3) diagnostic algorithms validated for chronic and congenital CD; (4) evidence generated on improved treatment options; and (5) market shaping and supply chain interventions to ensure equitable access to innovative products. Figure 1 demonstrates the project´s theory of change, a methodology used to describe the pathway towards the desired goal. 

**FIGURE 1:**
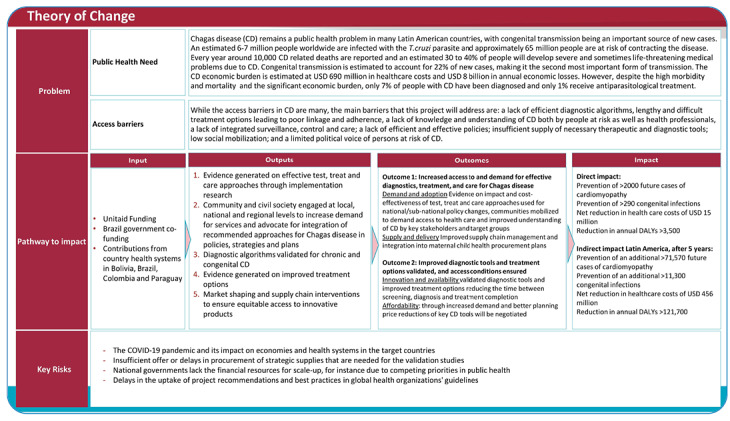
Theory of change.

In order to achieve Outputs 1 and 2, the project will conduct mixed-method implementation research using qualitative and quantitative approaches, which will be implemented in a total of 32 municipalities in Bolivia (10), Brazil (5), Colombia (12) and Paraguay (5) (Figure 2). These municipalities were selected according to public health priorities, ensuring geographically and epidemiologically diverse contexts, with primary health care (PHC) as the central focus of interventions, integrating with existing initiatives such as health and vaccination campaigns and the EMTCT-plus program. A great diversity of methods for diagnosing congenital CD has been described and recognized, which demands the generation of evidence in different endemic contexts^11^.

**FIGURE 2:**
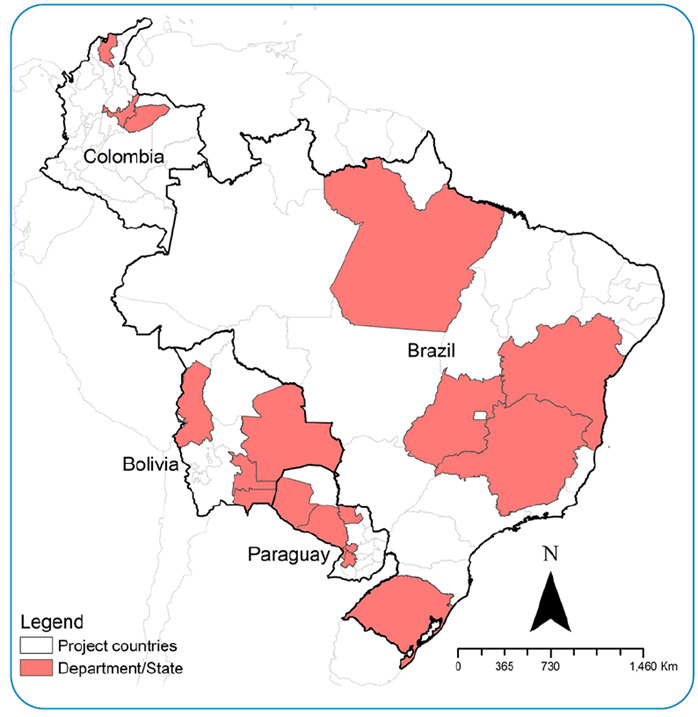
Target countries and its territorial entities participating in the CUIDA Chagas project.

The most cost-effective strategy to reduce the number of cases of congenital CD is the etiological treatment of WCBA before pregnancy^2^ ^,^ ^4^ ^,^ ^5^ ^,^ ^7^ ^-^ ^9^ ^,^ ^11^. In this regard, approximately 234,000 WCBA, their infants and children and their household contacts will be actively and systematically tested throughout the duration of the study (48 months). This study will conduct test, treat and comprehensive care interventions that will be integrated into PHC and MCH, and will include, amongst others, counseling services, the use of rapid diagnostic tests (RDTs) for screening to expedite the diagnostic process of chronic CD, use of molecular biology to facilitate an early diagnosis of CD in newborns, and provision of treatment courses. 

Formative studies will be carried out in each territory in order to better understand the local context, design context appropriate interventions, assess the performance of the local health system, the influence of socio-demographic descriptors, and the existence of systemic and psychosocial barriers. Health professionals will be trained in the use of (new) diagnostic algorithms and treatment schemes, as well as CD surveillance, clinical management and counseling, parasitological diagnosis, and molecular biology. The ‘test, treat and care’ service provision will be piloted and a monitoring and evaluation protocol specific to the implementation research will be developed in order to demonstrate the effectiveness of each intervention. A cost-effectiveness study will compare the interventions with the status quo in each country, considering their effects on health (disability adjusted life years (DALYs) and quality-adjusted life years (QALYs)). Through this comprehensive and integrated approach, contextualized implementation models will be provided and tested in different countries and will be made available for replication and scale-up.

Civil society (CS) is critical for both the success of the project as well as the sustainability of the outcomes, which is why they are engaged from the beginning through community advisory boards and specific project activities. Contextualized information, education and communication strategies and campaigns will be developed for different territories and target groups. Local leaders will be trained on the detection of CD signs and symptoms, potential adverse reactions of treatment, and the need for referral to PHC posts. Leadership training with community leaders and CS organizations representatives will be conducted on interrelated modules with the aim of strengthening their capacity to represent their communities and influence policies. 

To achieve Outputs 3 and 4, innovation protocols have been developed for three of the four countries included in this consortium (Bolivia, Brazil and Colombia) with the aim of overcoming barriers to obtain diagnosis and treatment. Diagnosing the different forms of CD is complex and results in limited access to treatment, for example, (i) chronic CD requires at least 2-laboratory based tests, and (ii) congenital CD diagnosis requires a diagnostic algorithm combining direct parasitological examinations at birth and two serological tests over a 9-12 months period, when there are no more antibodies from the infected mother. For chronic CD, RDTs provide only screening information and have not been widely implemented in public health systems in LA. 

Evidence suggests that RDTs could simplify algorithms for the diagnosis of chronic CD in PHC^2^ ^,^ ^7^ ^,^ ^8^; however, this requires further validation. The project will therefore conduct a study to demonstrate that RDT-based algorithms (single or multiple tests) can be implemented to diagnose chronic CD at PHC facilities, as an alternative to the current (laboratory-based) diagnostic algorithms, considering *T. cruzi* genetic variability and epidemiological diversity in CD endemic regions. 

Diagnosis of CD in newborns is complex and usually only possible at the end of the first year of life^4^ ^,^ ^5^ ^,^ ^7^ ^,^ ^8^. This leads to loss of follow-up, as many families do not return to the health center. Therefore, the project will implement a new algorithm in the pilot ‘test, treat and care’ service provision for the diagnosis of newborns based on polymerase chain reaction (PCR). Access to PCR in the first trimester of life will most likely reduce loss from follow-up and allow for more treatment.

An important barrier in reducing the CD burden is related to current treatment regimens, which are lengthy (60 days) and include frequent side effects, causing approximately 20% of patients to drop out of treatment while discouraging others from starting^2^ ^,^ ^12^. A shorter treatment regimen has the potential to greatly increase treatment adherence, which is why this project will conduct a double-blind, phase III study in which patients will be randomly assigned to receive the standard dose of benznidazole (300 mg daily for 60 days) or a short experimental regimen (300 mg daily for 2 weeks). Efficacy will be assessed by considering a non-inferiority design and the detection of parasite DNA using PCR. Meanwhile, safety will be evaluated through a superiority design, with the aim of finding a new regimen as effective as the standard one but superior in terms of safety. The study population will include adult patients who have been diagnosed with chronic CD in its indeterminate or mild cardiac form, and who have received a positive diagnosis through two serological assays. The primary endpoint will be parasitological response, determined as sustained negative qualitative PCR from the end of treatment until 24 months of follow-up. 

For Output 5, product landscape assessments and other market preparatory activities will be conducted to inform the development of product roadmaps that contain manufacturing/supply-side information, suggested go-to-market strategies in each country, as well as regulatory pathway and procurement strategies. All the proposed (innovation) strategies aim to shorten the time required for diagnosis and treatment, thereby lowering costs, and making strategies more accessible and cost-effective. This will increase access to dedicated PHC, covering a larger population, reducing losses during follow-up, and increasing case detection, while simultaneously providing patients with proper care.

To contribute to the scalability of the initiatives, regional collaboration will be strengthened and stimulated, not only between countries included in this project, but also with other countries endemic for CD and those where CD forms a public health challenge. A collaboration platform will be established to provide template protocols, communication and advocacy materials, lessons learned, and all relevant tools for non-project countries to consult or adapt in an effort to catalyze the project’s scope. 

By the end of the project, CUIDA Chagas is expected to have screened 234,000 people for CD, including 181,000 WCBA, 37,000 children and infants, and 16,000 other household members. Based on the prevalence of disease in the different communities participating in the project, this would enable treatment of 8,600 people (after accounting for contraindications and loss to follow-up). The direct impact would be the potential avoidance of 2,000 cases of cardiomyopathy and 290 congenital infections, with an annual reduction in healthcare costs of US$ 15 million and a decrease in annual DALYs of 3,500. In addition, CUIDA Chagas will provide implementation models that may be replicated in different contexts, as well as improved diagnostic and treatment options, all with the aim to increase access to CD diagnosis, treatment, and care for those that need it the most, contributing to achieving sustainable development goals for 2030^13^.
